# CTCF Prevents the Epigenetic Drift of EBV Latency Promoter Qp

**DOI:** 10.1371/journal.ppat.1001048

**Published:** 2010-08-12

**Authors:** Italo Tempera, Andreas Wiedmer, Jayaraju Dheekollu, Paul M. Lieberman

**Affiliations:** 1 The Wistar Institute, Philadelphia, Pennsylvania, United States of America; 2 Istituto Pasteur – Fondazione Cenci Bolognetti, Rome, Italy; University of North Carolina at Chapel Hill, United States of America

## Abstract

The establishment and maintenance of Epstein-Barr Virus (EBV) latent infection requires distinct viral gene expression programs. These gene expression programs, termed latency types, are determined largely by promoter selection, and controlled through the interplay between cell-type specific transcription factors, chromatin structure, and epigenetic modifications. We used a genome-wide chromatin-immunoprecipitation (ChIP) assay to identify epigenetic modifications that correlate with different latency types. We found that the chromatin insulator protein CTCF binds at several key regulatory nodes in the EBV genome and may compartmentalize epigenetic modifications across the viral genome. Highly enriched CTCF binding sites were identified at the promoter regions upstream of Cp, Wp, EBERs, and Qp. Since Qp is essential for long-term maintenance of viral genomes in type I latency and epithelial cell infections, we focused on the role of CTCF in regulating Qp. Purified CTCF bound ∼40 bp upstream of the EBNA1 binding sites located at +10 bp relative to the transcriptional initiation site at Qp. Mutagenesis of the CTCF binding site in EBV bacmids resulted in a decrease in the recovery of stable hygromycin-resistant episomes in 293 cells. EBV lacking the Qp CTCF site showed a decrease in Qp transcription initiation and a corresponding increase in Cp and Fp promoter utilization at 8 weeks post-transfection. However, by 16 weeks post-transfection, bacmids lacking CTCF sites had no detectable Qp transcription and showed high levels of histone H3 K9 methylation and CpG DNA methylation at the Qp initiation site. These findings provide direct genetic evidence that CTCF functions as a chromatin insulator that prevents the promiscuous transcription of surrounding genes and blocks the epigenetic silencing of an essential promoter, Qp, during EBV latent infection.

## Author Summary

Epstein-Barr Virus (EBV) establishes a latent infection that is associated with several lymphoid and epithelial cell malignancies. The latent virus persists as a circular minichromosome in the nucleus of infected cells. Epigenetic modifications of the viral DNA and chromatin are known to control viral gene expression and genome stability, but the nature and mechanisms of these epigenetic marks are not known. Here, we use viral genome-wide analysis to characterize patterns of DNA and histone methylation, and how these are organized by the chromatin boundary factor CTCF. Mutation of one such CTCF site at the EBV Q promoter results in aberrant accumulation of DNA CpG methylation and histone H3 K9 trimethylation, and the consequent silencing of Qp transcription. We conclude that CTCF chromatin insulator function is required for the epigenetic programming and stable maintenance of latent viral infection.

## Introduction

Epstein-Barr Virus (EBV) is a human gamma herpesvirus that establishes latent infection in more than 90% of the adult population world-wide [1], [2]. The ∼170 kb genome encodes ∼90 viral genes but only a few of these are expressed during latent infection. The latent infection is a cofactor in several human malignancies and may play an essential causative role in the endemic forms of Burkitt's lymphoma (BL) and nasopharyngeal carcinoma (NPC), as well as diffuse B-cell lymphomas in HIV-AIDS and iatrogenic immunosuppressed individuals [3]. Remarkably, the viral gene expression patterns vary in each tumor type suggesting that EBV can establish multiple forms of latency [4]. These different gene expression programs have been referred to as latency types and may also correlate with the changes in host-cell differentiation state and tissue origin [4], [5]. Changes in EBV latency type may also be important for evasion of host-immune recognition [6].

EBV latency gene expression programs have been categorized into four different types based primarily on the differential expression of the EBNA and LMP gene transcripts [4]. Type 0 latency is defined as the absence of expression of any viral genes, and is thought to exist in quiescent memory B-cells [5], [7]. Type I latency is characterized by the expression of the EBNA1 gene only, and is observed in proliferating memory B-cells in normal hosts, and found predominantly in Burkitt lymphoma tissue and derived cell lines [8], [9], [10]. Type II latency is characterized by the expression of EBNA1 and LMP2 expression, with some variable expression of LMP1. This pattern of gene expression is observed in epithelial cell derived tumors including NPC and gastric carcinomas [11], [12], [13]. Type III latency is characterized by the expression of EBNA-1, -2, -3A, -3B, -3C,-LP, LMP1, LMP2. This more permissive gene expression program is observed upon primary infection of naïve B-cells and is associated with B-cell proliferation and immortalization [14]. Type III latency is observed in immortalized B-cells in culture and diffuse B-cell lymphomas in immunosuppressed individuals. The natural history of EBV infection suggests that type III latency progresses to type I latency during B-cell maturation, and that viral lytic occurs in terminally differentiating plasma B-cells [5], [15].

Latency type gene expression is regulated largely through differential promoter utilization [16], [17], [18], [19], [20]. The promoters for type III infection are activated upon primary infection by B-cell specific factors [21]. Initial transcription from Wp promoter allows the expression of EBNA2, which functions as a transcriptional activator or the Cp promoter which drives expression of EBNA-LP, EBNA-2, EBNA-3A, -3B, -3C and EBNA1 [22], [23], [24], [25]. EBNA2 also activates LMP1 and LMP2 transcription to maintain type III gene expression [25]. Type II and Type I gene expression arise through mechanisms that are not completely understood, but involve the epigenetic silencing of the Cp and LMP1 promoter by DNA methylation and histone deacetylation [26], [27], [28], [29], [30], [31], [32], [33]. EBNA1 expression is required for the replication and maintenance of the viral latent genome [34], [35], [36], [37]. EBNA1 mRNA expression is maintained in type III latency by Cp promoter utilization and mRNA processing, while in type I latency EBNA1 expression is driven largely through the Q promoter (Qp) [17], [31], [38], [39], [40]. EBNA1 protein binds to two sites located at the +10–+57 position relative to the Qp transcription initiation site and restrict its usage in type III cells where EBNA1 proteins levels are elevated [41], [42]. Thus, EBNA1 can autoregulate its own expression levels through promoter selection, and help to coordinate the switch between latency types.

The epigenetic control of EBV promoter utilization and latency type is evident in the differential pattern of DNA methylation between latency types, and by the ability of DNA methylation inhibitors to stimulate type III gene transcription from type I latently infected cells [26], [43]. It is also apparent that histone acetylation occurs at the Cp promoter during type III latency where they are transcribed, but not in type I latency where Cp promoter is silenced [44]. However, little else is known about the epigenetic controls the determine promoter utilization and gene expression during the different latency types. LMP1 and Cp activation depend on the enhancer functions of EBNA2 and EBNA1. EBNA1 binds to the EBV origin of plasmid (OriP) replication and is essential for both viral replication and plasmid maintenance, as well as for transcriptional enhancement of EBNA2 and LMP1 [45], [46], [47]. The mechanism through which EBNA1 activates Cp and LMP1 from OriP, which is located over 2 kb from each promoter is not clear. It is also not known whether EBNA1 binding at Qp may also regulate transcription of type III promoters.

Cellular factors that regulate communication between promoters and enhancers, have also been implicated in the organization of chromatin structure [48], [49], [50], [51]. The chromatin insulator protein CTCF has been implicated in segregating active from inactive chromatin domains, as well as in mediating long-distance interactions between transcriptional regulatory regions [52], [53]. At least one CTCF site has been mapped to a region between OriP and Cp, and its binding was found to correlate with the inhibition of Cp transcription in type I latency [54], [55]. Other CTCF sites in EBV chromosome are known to exist, but their function has not been explored in detail [54]. In this work, we use a genome-wide ChIP assays to explore the epigenetic landscape of the EBV genome in type I and type III infected cells. We found that CTCF binding sites are positioned in key regulatory locations throughout the viral genome. We investigate in detail the function of a high affinity CTCF site positioned immediately upstream of the EBNA1 binding sites in Qp. We find that CTCF binding is required to maintain the transcriptional activity and prevent the epigenetic silencing of Qp in proliferating cells.

## Results

### Epigenetic differences between type I and type III genomes

**Figure 1:**
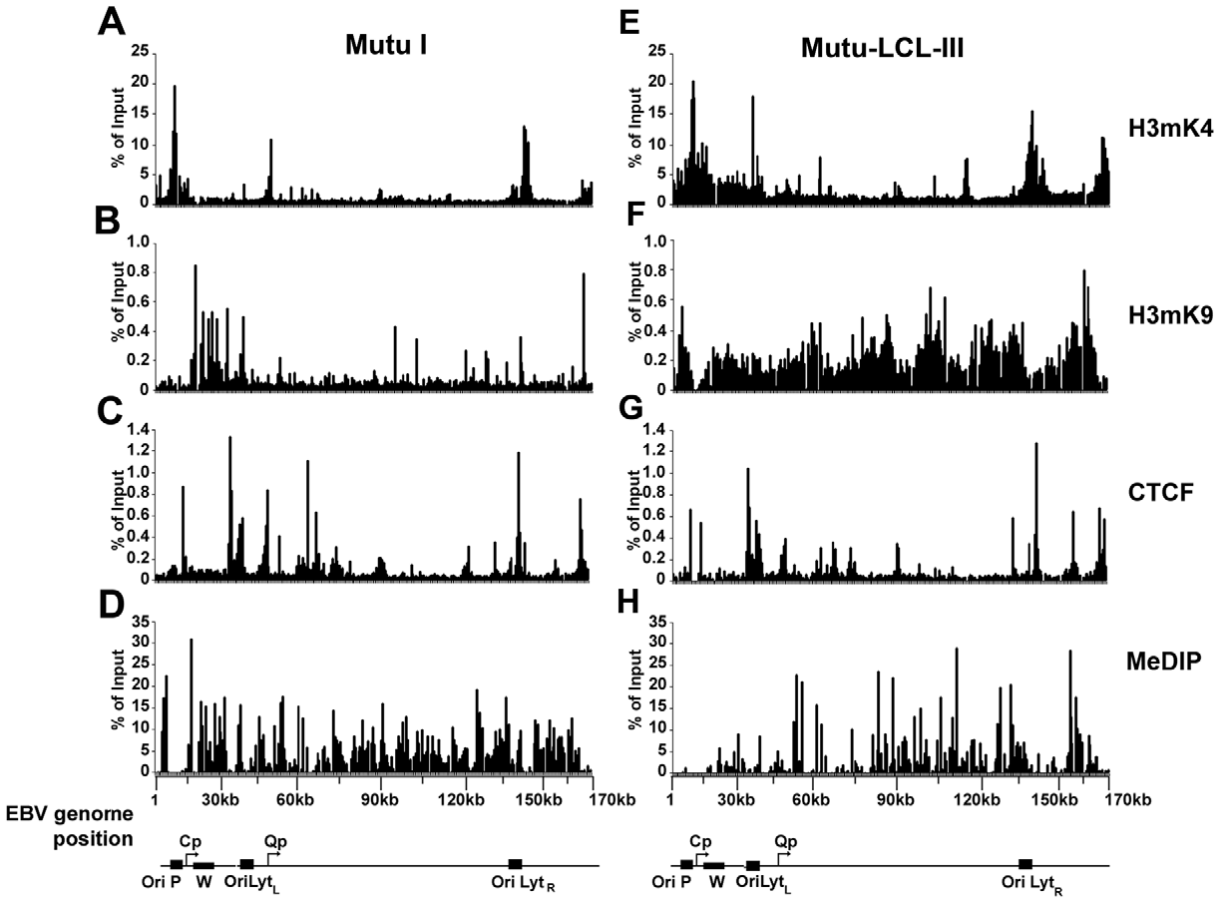
EBV genome-wide analysis of histone and DNA methylation patterns in different latency types. ChIP assays were performed with Mutu I (A–D), or Mutu-LCL (E–H) using antibodies for histone H3me2K4 (A and E), H3me3K9 (B and F), CTCF (C and G), or methyl cytosine (MeDIP) (D–H). ChIP DNA was assayed by real-time PCR using a genome wide array of 384 primers spaced ∼400 bp across the EBV genome. Approximate EBV genome positions are indicated in the schematic below each column of graphs. Graphs represent an average of three independent experiments. Standard deviation was less than 10 percent of the mean for all data points.

An EBV genome-wide real-time PCR array was used to compare the patterns of several epigenetic marks between type I and type III latent virus genomes using the chromatin immunoprecipitation (ChIP) assay (Fig. 1). For these experiments we used a 384-well array that covers the entire EBV genome at a density of ∼400 bp between primer sets. In a previous study, we used a similar approach to analyze the first 60 kb of the EBV genome for various histone modifications and protein factor binding sites [54]. In the present study, we compared a type I latently infected Burkitt lymphoma cell line, Mutu I, with a lymphoblastoid cell line derived from Mutu I viral DNA (Mutu-LCL), ensuring that these two cell types were isogenic with respect to EBV genomes. We compared the pattern of CTCF binding sites to those of histone H3me3K9 (H3mK9) and H3me2K4 (H3mK4) modifications and also that of cytosine methylation (mCpG) using methyl DNA immunoprecipitation (MeDIP). Several patterns were noteable. Peaks of histone H3mK9 and H3mK4 were complementary and non-overlapping. Major peaks of H3mK4 were detected at the regions surrounding the RNA polymerase III transcribed EBERS and the Bam A microRNA cluster in both cell types. H3mK4 peaks regions surrounding the LMP1 promoter and Cp promoter were elevated in type III relative to type I cells. H3mK9 methylation was elevated over the Cp and W repeats in type I latency, but not type III, correlating with transcription repression in type I. CTCF binding sites were located at multiple positions, with only a few differences in type I and type III. CTCF sites tended to exist between clusters of H3mK4 and H3mK9, as can be seen at a newly discovered peak at the region 3′ of the W repeats. All the CTCF binding sites found in our assay are listed in Table S1. mCpG patterns were also different between type I and III latency. High levels of mCpG were detected across the Cp and LMP2 regions in type I, but not in type III, correlating with transcription silencing of Cp/Wp in type I cells. Interestingly, CTCF sites tended to demarcate boundaries of mCpG, which also correlated well with H3mK9 in both cell types.

**Figure 2:**
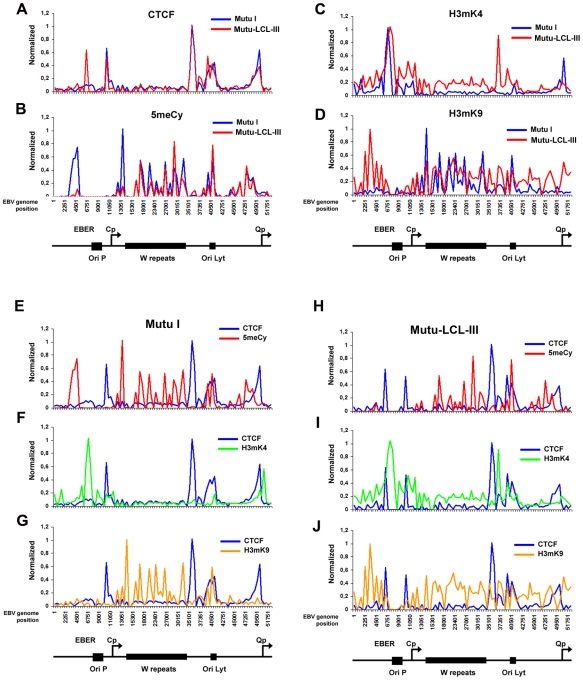
Analysis of epigenetic patterns in the EBV latency control region. A–D) EBV genome-wide ChIP data from figure 1 was reanalyzed at EBV positions 1–60 kb as direct comparison between Mutu I (blue) and Mutu-LCL (red) for CTCF (panel A), methyl cytosine (panel B), H3me2K4 (panel C), H3me3K9 (panel D). E–G) Mutu I cells compared for CTCF (blue) vs methyl cytosine (red); CTCF (blue) vs H3me2K4 (green); CTCF (blue) vs H3me3K9 (yellow). H–J) Mutu-LCL-III cells compared for CTCF (blue) vs methyl cytosine (red); CTCF (blue) vs H3me2K4 (green); CTCF (blue) vs H3me3K9 (yellow).

A more detailed examination of the regions surrounding the major latency promoter elements reveals other features relevant to epigenetic regulation (Fig. 2). While most CTCF sites are bound similarly in each cell type, the CTCF site upstream of the EBERS was significantly reduced in Mutu I cells (Fig. 2A). Interestingly, this region is enriched in mCpG in MutuI, relative to type III cells (Fig. 2B). One possible explanation for the loss of CTCF binding at this region in type I cells is that enriched DNA methylation replaces and blocks CTCF binding. In contrast, this same region is elevated in H3mK9 in type III latency, where CTCF occupies a 3′ boundary upstream of the EBERS (Fig. 2D and 2J). Another striking feature is the elevated H3mK4 across the EBNA2 transcript and BHRF1 miRNA cluster in type III latency, but not in type I (Fig. 2C). This correlates well with the difference in RNA polymerase II transcription across this region in these two cell types. The CTCF site at this position just 3′ of W repeats provides a 5′ border for the high H3mK4 in type III latency (Fig. 2I), and a 3′ border for the high H3mK9 (Fig. 2E) and mCpG (Fig. 2G) in type I latency.

CTCF binds consistently at Qp in both cell types (Fig. 2A). In type I cells, the Qp CTCF site separates a 3′ H3mK4 peak from a 5′ region enriched in H3mK9 and mCpG (Fig. 2E–G). In type III cells, the CTCF site appears to spare Qp from surrounding regions of elevated mCpG and H3 mK9 (Fig. 2H and J). The region is also reduced in H3mK4, corresponding to a reduction in EBNA1 binding and transcription from Qp (Fig. 2I). These marks are largely consistent with known transcription properties of Qp in which it is active in type I, and repressed in type III (Figure S1).

### Mapping the CTCF site at Qp

**Figure 3:**
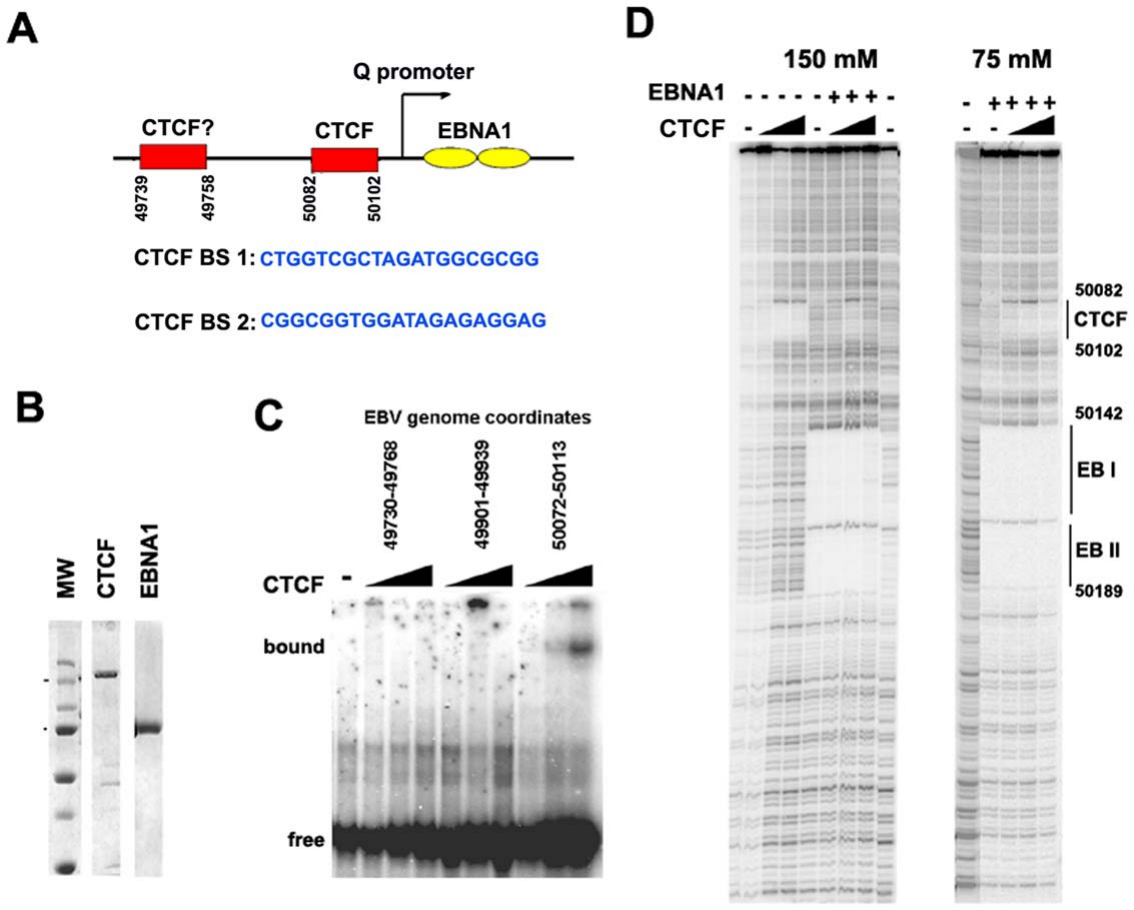
Identification of a CTCF binding site upstream of Qp. A) Schematic of CTCF and EBNA1 binding site organization at Qp, and the sequence of candidate CTCF binding sties (BS) 1 and 2. B) Coomassie stain of purified recombinant CTCF and EBNA1 proteins derived from baculovirus expression system. C) EMSA analysis of purified CTCF (10–100 ng) binding to DNA probes for EBV regions 49730–49768 (BS1), 49901–49939, or 50072–50113 (BS2). D) DNase I footprinting assay of purified CTCF protein (30–300 ng) in the absence (-) or addition (+) of 30 ng purified EBNA1, in buffer containing 150 mM (left panel) or 75 mM (right panel) NaCl.

To identify the specific sequence element bound by CTCF near Qp, we first examined the region for candidate CTCF binding sites using a prediction algorithm (http://insulatordb.uthsc.edu). At least two candidate CTCF sites were identified at positions 43739 and 50082 (Fig. 3A). These sites were synthesized as DNA oligonucleotide probes and tested by EMSA for binding to purified recombinant CTCF protein (Fig. 3B and C). We found that CTCF bound efficiently to the 50082 binding site, but not to the 49739 sequence or to a control oligonucleotide (from EBV 49901) that lacked any candidate CTCF binding site (Fig. 3C). The precise nucleotide binding site of CTCF was mapped by DNase I footprinting using the entire Qp control region as a probe and purified recombinant CTCF (Fig. 3D). We found that CTCF protected a ∼20 bp regions between 50082–50102. In the same DNase footprinting reaction we included recombinant EBNA1 protein. EBNA1 bound to two sites in Qp covering EBV nucleotides 50142 to 50189.

The DNase I footprinting assays demonstrate that CTCF and EBNA1 can bind simultaneously to Qp, and that potential interactions between these proteins may regulate Qp. However, detailed biochemical analysis of these interactions were limited by the different salt sensitivities of the purified proteins in the DNA binding assays (Fig. 3D). The physiological significance of this differential salt sensitivity is not clear.

### Generation of an EBV Bacmid with CTCF mutations at Qp

**Figure 4:**
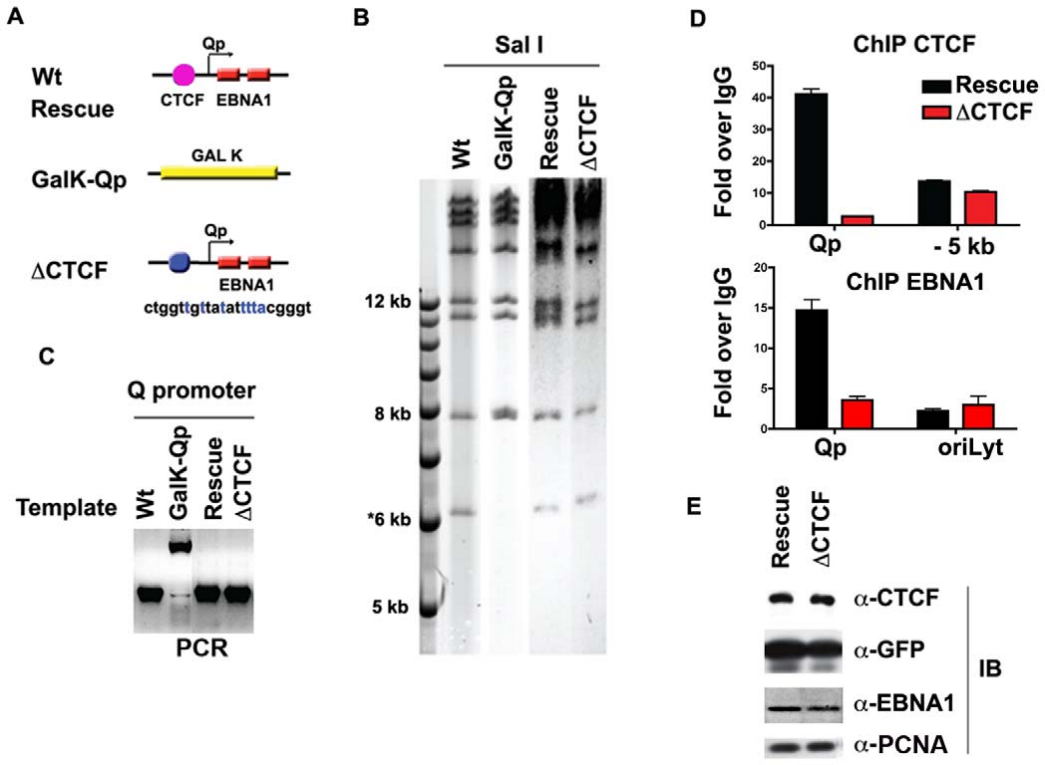
Mutagenesis of CTCF binding site in Qp. A) Schematic of mutations introduced into the Qp region of EBV bacmid. B) Purified bacmid DNA for EBV Wt, GAL K, Wt rescue, and ΔCTCF was analyzed by Sal I restriction digest and 0.7% agarose gel electrophoresis. DNA was visualized by ethidium bromide staining. C) PCR amplification of the region encompassing Qp for EBV Wt, GAL K, Wt rescue and ΔCTCF. D and E) ChIP assay of Wt rescue, or ΔCTCF bacmids in stable 293 cell pools after 8 weeks of hygromycin selection with antibody for CTCF (top panel), or EBNA1 (lower panel). CTCF ChIP was analyzed at Qp or a region −5 kb to Qp. EBNA1 ChIP was analyzed at Qp, or at OriLyt control region. E) Western blot analysis of CTCF, GFP, EBNA1, and PCNA protein levels for Wt rescue or ΔCTCF 293 cell pools.

To investigate the functional significance of CTCF binding at Qp in cell-based assays, we engineered a substitution mutation in the CTCF binding site at Qp in EBV bacmids using recombineering with GALK gene insertion and gene replacement [56] (http://recombineering.ncifcrf.gov) (Fig. 4A). GALK insertion, CTCF substitution mutation (ΔCTCF), and Wild-type (Wt) rescue mutants in EBV bacmids were validated by restriction enzyme digestion (Fig. 4B), PCR across the junctions (Fig. 4C) and sequencing of the insertions (data not shown). Bacmid DNA for ΔCTCF and Wt rescue control was introduced into 293 cells and stable transformants were selected for hygromycin and GFP expression. After 8 weeks of selection, stable cell pools were assayed by ChIP assay to validate that the substitution mutation disrupted CTCF binding in living cells (Fig. 4D). As expected, CTCF failed to bind to Qp in the ΔCTCF mutant (Fig. 4D, top panel). We also found that EBNA1 binding to Qp was reduced in the ΔCTCF mutant (Fig. 4D, lower panel), suggesting that CTCF facilitates EBNA1 binding at Qp in living cells. Stable cell pools were also assayed at 8 weeks for their relative expression GFP, EBNA1, CTCF and PCNA (Fig. 4E). We found no apparent differences in the expression of these proteins after 8 weeks of selection in 293 cell pools.

### CTCF binding site at Qp is required for stable maintenance of EBV episomes in 293 cells

**Figure 5:**
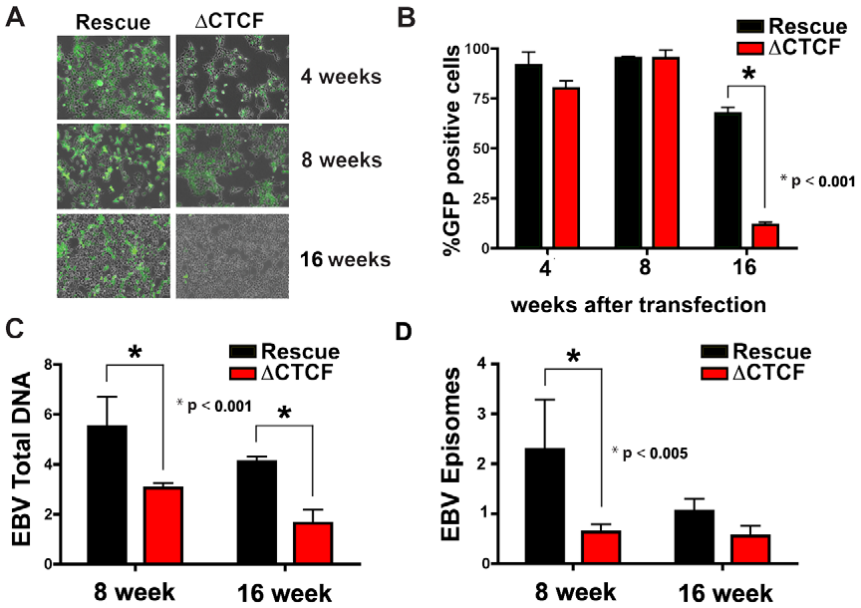
CTCF binding site at Qp is required for stable maintenance of EBV episome in 293 cells. A) Photomicrographs of GFP fluorescence of EBV bacmid Wt rescue or ΔCTCF in 293 cell pools after 4, 8 and 16 weeks post-transfection. B) EBV episome maintenance in Wt rescue or ΔCTCF 293 cell pools was assayed by FACS analysis as the percentage of GFP positive cells at the indicated weeks. Bars represent the average of three independent experiments. C) EBV genome copy number was assayed by real time PCR analysis in Wt rescue or ΔCTCF 293 cell pools at the indicated weeks. Bars represent the average of three independent experiments. D) Episomal viral DNA from Wt rescue or ΔCTCF 293 cell pools was isolated at the indicated weeks by Hirt extraction and assayed by quantitative real time PCR using the viral DNA from Raji cells as reference (ΔΔCt method). Bars represent the rate of episome lost of three independent experiments. All error bars indicate the standard deviation from the mean.

While early passage cell pools showed little difference in GFP and EBNA1 expression between ΔCTCF and Wt rescue genomes, we observed a marked loss of GFP expression in the ΔCTCF relative to Wt rescue genomes after longer passages in culture (Fig. 5A and B). At 4 and 8 weeks after transfection, ΔCTCF and Wt rescue had nearly identical percentage of GFP positive cells. In contrast, at 16 weeks ΔCTCF pools were ∼9% positive, while Wt rescue was ∼72% GFP positive, as measured by FACS (Fig. 5B). EBV DNA copy number per cell was determined by real time PCR for 293 cell pools at 8 and 16 weeks (Fig. 5C). We found that ΔCTCF cell pools had ∼50% less EBV DNA per cell than Wt rescue containing pools as measured at 8 and 16 weeks. Isolation of EBV episomes by Hirt lysis revealed that ΔCTCF cell pools had ∼3 fold lower DNA than WT rescue at 8 weeks, and both pools were reduced at 16 weeks, indicating that ΔCTCF episomes are lost at a greater rate than Wt episomes (Fig. 5D). These data suggest that the Qp CTCF binding site is important for the stable maintenance of EBV episomes in selected 293 cell pools.

### ΔCTCF genomes have altered transcription and promoter usage

**Figure 6:**
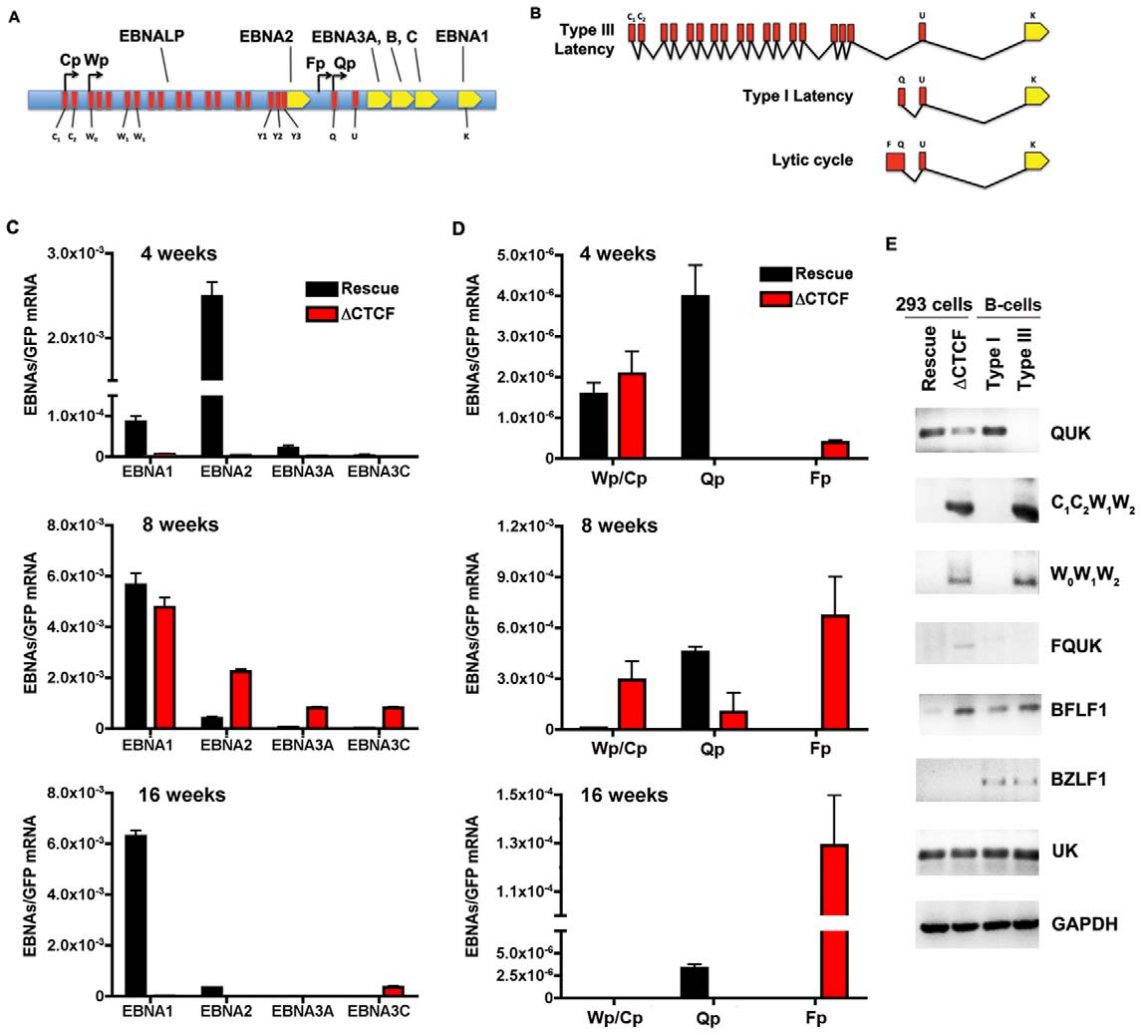
RNA expression and promoter utilization in Qp mutated bacmids. A) Schematic representation of the EBV latency genes and promoters. Promoters are indicated by arrows. The position of the six EBNAs ORFs are indicated. B) Schematic representation of different EBNA1 transcripts. Exons present at 5′ end of EBNA1 mRNA are indicated in red. C) Quantitative RT-PCR was used to measure the abundance of EBNA2, EBNA3A and EBNA3C mRNA relative to bacmid GFP for Wt rescue or ΔCTCF bacmids in 293 cell pools at 4, 8, and 16 weeks after transfection, as indicated. D) Same as in C, except EBNA1-transcripts initiating from either Cp/Wp, Qp, or Fp were measured relative to GFP in Wt rescue or ΔCTCF bacmids in 293 cell pools at 4, 8, and 16 weeks after transfection. E) RT-PCR was measured for Wt rescue or ΔCTCF bacmids in 293 cell pools at 8 weeks post-transfection, as well as for type I (Mutu I) or type III (Mutu-LCL) controls. RNA was analyzed for the junction specific transcripts QUK (Qp initiation), C_1_C_2_W_1_W_2_ (Cp initiation), W_0_W_1_W_2_ (Wp initiation), BFLF1 (lytic gene adjacent to Qp), UK (EBNA1 mRNA in both type I and type III), and control cellular GAPDH.

The loss of GFP expression and episome stability in 293 cell pools could be due to a deregulation of viral gene expression. Others have shown that EBV establishes a restricted pattern of latency gene expression in 293 cells, resembling a type I program with stable Qp utilization for EBNA1 expression [57]. To assess viral gene expression patterns in transfected 293 cells, we first assay mRNA expression of EBNA1, EBNA2, EBNA3A, and EBNA3C in 293 cell pools after 4, 8, and 16 weeks of selection (Fig. 6). RNA expression was measured by quantitative RT-PCR and normalized to bacmid expression of GFP mRNA. At 4 weeks, EBNA1 and EBNA2 mRNA levels were expressed at lower levels in ΔCTCF compared to Wt rescue cell pools (Fig. 6C, top panel). At 8 weeks, EBNA1 levels were similar, while EBNA2, EBNA3A, and EBNA3C levels were higher in ΔCTCF relative to Wt rescue cell pools (Fig. 6C, middle panel). By 16 weeks, EBNA1 mRNA levels were maintained in the Wt rescue, but almost undetectable in ΔCTCF cell pools (Fig. 6C, lower panel). EBNA2, EBNA3A, and EBNA3C were expressed at very low levels in both Wt rescue and ΔCTCF cell pools at these later passages in culture. These observations are consistent with a previous study showing that EBV initially expresses EBNA2, but eventually adopts an EBNA1 only, type I latency in 293 cells [57].

To better understand the failure of ΔCTCF bacmids to sustain EBNA1 mRNA expression, we investigated the promoter utilization at 4, 8, and 16 weeks after transfection (Fig. 6D). EBNA1 mRNA has been shown to initiate from Wp/Cp in most type III latency, from Qp in most type I latency, and from Fp during lytic cycle gene expression (Fig. 6A and B). We assayed the utilization of Wp/Cp, Qp, and Fp using quantitative RT-PCR with primers specific for each promoter (Table S2). At 4 weeks, we found that Wp/Cp was utilized at similar levels in Wt rescue and ΔCTCF containing cell pools (Fig. 6D, top panel). Remarkably, Qp was utilized at relatively high levels in Wt rescue, but nearly undetectable in ΔCTCF. Interestingly, Fp utilization was detected in ΔCTCF, but undetectable in Wt rescue. At 8 weeks, Wt rescue containing cells utilized Qp predominantly, while ΔCTCF cells utilized all three promoters, with Fp dominating (Fig. 6D, middle panel). By 16 weeks, Wt rescue genomes utilized Qp exclusively, while ΔCTCF genomes utilized Fp exclusively, although ∼ 10 fold less than Fp utilization at 8 weeks (Fig. 6D, lower panel). Since Fp is typically associated with lytic gene activity, we tested whether ΔCTCF containing cells were expressing the lytic immediate early gene BZLF1 (Fig. S3 and Fig. 6E). BZLF1 expression was undetectable at all time points tested for Wt or ΔCTCF 293 cell pools, as measured by quantitative RT-PCR (Fig.S3) or by conventional PCR (Fig. 6E). These findings suggest that latency promoter utilization is deregulated in ΔCTCF genomes.

To better understand the ΔCTCF defects in viral gene expression, we assayed viral mRNA using exon specific PCR for transcripts (Table S3), initiating at Qp (QUK), Cp (C_1_C_2_W_1_W_2_), Wp (W_0_W_1_W_2_), or within the lytic transcripts of BFLF1 [58] (Fig. 6E). In addition, we measured the UK intron junction for EBNA1, which is expressed in type I and type III cells. We also measure BZLF1 expression as an indicator of lytic cycle gene expression. As a control we assayed the expression levels of cellular GAPDH. RNA was isolated from pools after 8 weeks in culture. We found that QUK was expressed at slightly higher levels in Wt rescue relative to ΔCTCF cell pools (Fig. 6E, top panel). C_1_C_2_W_1_W_2_, W_0_W_1_W_2_ and FQUK transcripts were elevated in ΔCTCF relative to Wt rescue, consistent with RT-PCR data showing elevated levels of Wp/Cp and Fp utilization in bacmid lacking the CTCF binding site. UK transcripts were similar in ΔCTCF and Wt rescue, consistent with observations from quantitative RT-PCR (Fig. 6C) showing that EBNA1 mRNA levels were similar at 8 weeks after transfection. BZFL1 was not detected in either bacmid 293 cell pool, indicating that lytic gene activation or DNA replication was not indirectly responsible for these differences in gene expression. These exon-specific PCR studies further substantiate the real-time PCR data, and support the conclusion that CTCF mutations in Qp deregulate the latency type transcription pattern and promoter utilization.

### CTCF binding site is required for the maintenance of epigenetic patterns at Qp

**Figure 7:**
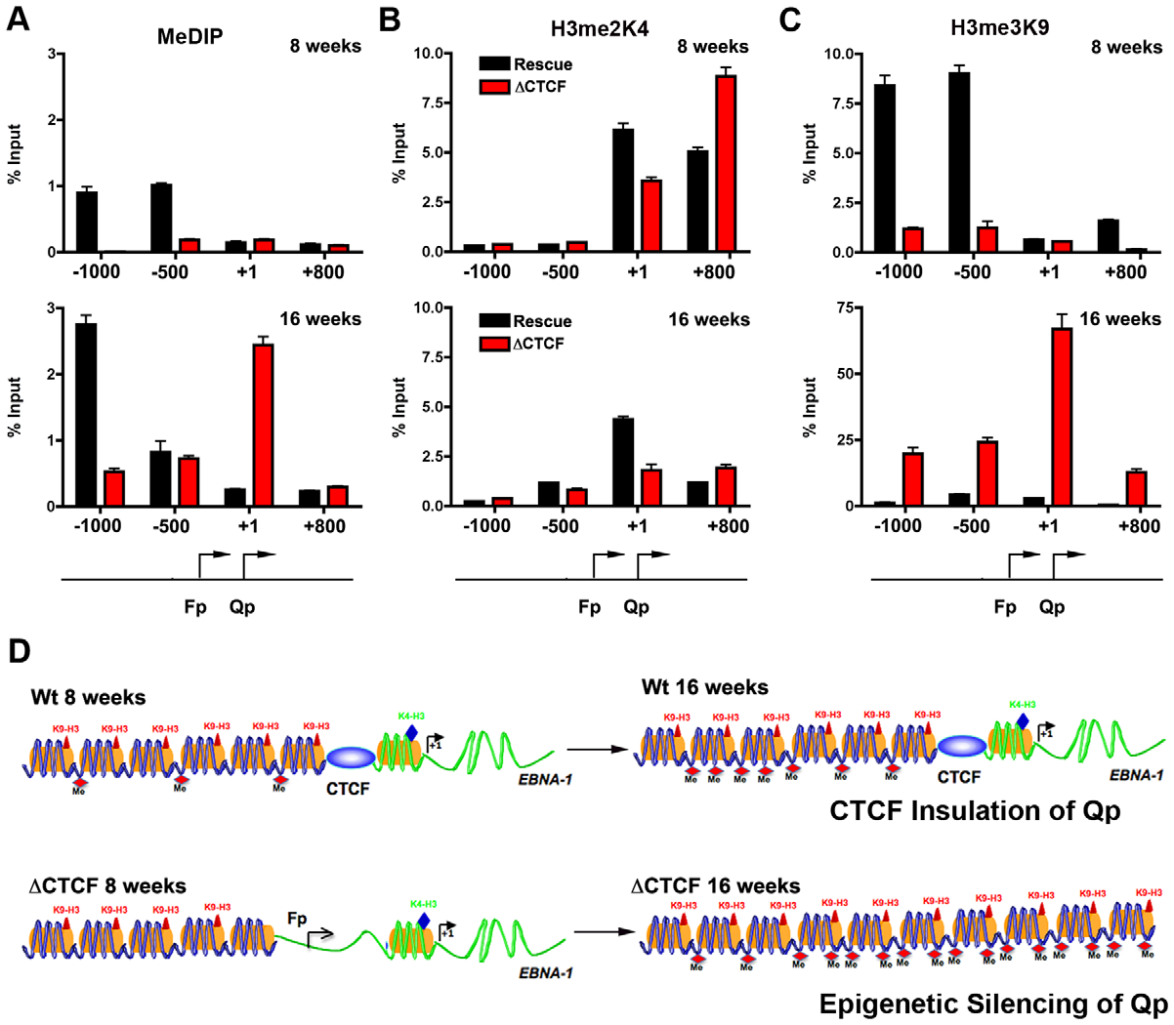
Changes in epigenetic patterns in Qp mutated bacmids. A) The epigenetic pattern of Qp region in Wt rescue and ΔCTCF bacmids in 293 cell pools was analyzed by MeDIP assay (A), H3me2K4 (B) and H3me3K9 (C) ChIp assay at 8 weeks (top panel) or 16 weeks (lower panel) after transfection. A schematic representation of Qp region is shown. D) Model of CTCF function in the chromatin organization at EBV Q promoter. CTCF is positioned as a barrier to the 5′ encroachment of H3me3K9 and mCpG in Wt rescue 293 cells (top panel). In ΔCTCF 293 cells, H3me2K4 is enriched throughout Qp and Fp at 8 weeks (lower panel, left), but is converted to H3me3K9 and mCpG at later times (16 weeks) (lower panel, right), indicating that CTCF is required to prevent this epigenetic drift at Qp.

CTCF has been implicated in several functions, including chromatin insulation and boundary functions. To determine if the disruption of CTCF site at Qp altered the normal pattern of epigenetic marks surrounding Qp, we performed MeDIP and ChIP assays with a set of primers that probe the regions −1000, −500, +1, and +800 relative to the Qp initiation site (Fig. 7A–C). At 8 weeks post-transfection, MeDIP revealed that mCpG was enriched at −1000 and −500 position in Wt rescue, but undetectable in ΔCTCF genomes (Fig. 7A, top panel). At 16 weeks post-transfection, mCpG was elevated at −1000 in WT rescue, but not at +1 or +800, consistent with high levels of transcription initiating at Qp in these cell pools (Fig. 6). In contrast, mCpG was elevated at +1 position in ΔCTCF genomes, consistent with the lack of Qp transcription initiation at 16 weeks in ΔCTCF cells (Fig. 6). To examine potential changes in euchromatic or heterochromatic histone modifications surrounding Qp, we focused on H3mK4 or H3mK9, respectively. At 8 weeks post-transfection, we found high levels of H3mK4 at the +1 and +800 positions in both Wt rescue and ΔCTCF genomes (Fig. 7B, top panel). At 16 weeks, H3mK4 was elevated primarily in Wt rescue (Fig. 7B, lower panel), consistent with persistent transcription from Qp in these cells (Fig. 6). The heterochromatic mark for histone H3mK9 was more revealing since it showed elevated levels upstream of Qp (−1000 and −500) in Wt rescue genomes at 8 weeks (Fig. 7C, top panel), and low levels in ΔCTCF where Fp is active. At 16 weeks, H3mK9 was highly enriched at +1 site of Qp in ΔCTCF genomes (Fig. 7C, lower panel), consistent with the transcription inactivity of Qp in these cells at this time after transfection. The epigenetic pattern surrounding Cp and Wp was also examined (Fig. S5). We found that Cp and Wp were hypermethylated and enriched on H3mK9 both at 8 and 16 weeks in Wt rescue, consistent with a previous study demonstrating that Cp and Wp undergo transcription silencing in 293 cells [57]. In ΔCTCF genomes Cp and Wp are hypomethylated and enriched on H3mK4 at 8 weeks, consistent with transcription initiation. However, by 16 weeks, Cp and Wp show elevated mCpG and H3mK9, consistent with the extinction of transcription initiation from these promoters at later passages. Thus, Cp and Wp undergo similar epigenetic silencing with Wt and ΔCTCF genomes, but Qp is protected from epigenetic silencing only in genomes were the CTCF site is intact (Fig. 7D).

## Discussion

Stable gene expression programs, like those associated with cell-type differentiation, correlate with heritable epigenetic changes to the cellular chromosome. The meta-stable gene expression programs associated with Epstein-Barr virus latency types have also been shown to correlate with epigenetic changes in the viral genome. The most consistently observed epigenetic difference between type I and type III latency is the DNA methylation of the Cp and LMP1 promoter regions [26], [43], [59]. We and others have also explored histone modification patterns at the major latency control regions for EBV, and observed distinct patterns between cell lines carrying EBV genomes with either type I and type III latency [44], [54], [60]. In this work, we extended this approach to examine the pattern of histone H3 K4 and K9 methylation, DNA methylation, and CTCF binding across the complete EBV genome in cells carrying stable type I or type III EBV latent infections (Figs 1 and 2, Figs. S1 and S2). We found that CTCF binding sites were located at or near regulatory regions, and commonly marked boundaries between euchromatic and heterochromatic marks (Fig. 2). The heterochromatic mark was typically H3mK9 in type III latency (e.g. EBV regions 2–6 kb in Fig. 2D), and mCpG, especially in type I latency (e.g. EBV regions 2–6 kb in Fig. 2B). This may reflect the natural history of epigenetic silencing where a partially repressive histone modification, like H3mK9 in type III, may eventually evolve into the more stable silencing modification associated with DNA methylation in type I. In some cases, the emergence of extensive CpG methylation correlates with the loss of CTCF binding (e.g. EBV region ∼6 kb in Fig. 2E and 2H). CTCF binding is known to be sensitive to CpG methylation [61]. These observations suggest that CTCF plays a key role in organizing epigenetic marks along the EBV genome, and that CTCF binding and epigenetic patterns change in different latency types.

To directly test the function of CTCF and other sequence specific binding factors in maintaining EBV latency type gene expression programs, we focused on the EBV Q promoter. We found that CTCF bound to Qp, and we used DNase I footprinting to map this binding site to a ∼20 bp region located ∼40 bp upstream of the EBNA1 binding sites (Fig. 3). In type I cells, CTCF appeared to separate a 3′ enrichment of H3mK4 that overlaps the Q transcription initiation site, from a 5′ enrichment of CpG methylation, that covers the neighboring lytic BFLF1 gene (Fig. 2E–G, Fig. S1). In type III cells, CTCF appears to spare the Qp transcription initiation site from surrounding H3 K9 methylation (Fig. 2I). To directly test the function of CTCF binding at Qp, we used recombineering methods to engineer a mutation that disrupts CTCF binding in EBV bacmids (Fig. 4). Disruption of CTCF binding site at Qp caused a loss of stable GFP expression and loss of bacmid episomes after multiple cell divisions (Fig. 5). CTCF site disruption also caused an increase in Fp promoter utilization, with no other evidence of lytic gene activation (Fig. 6C–E). Consistent with changes in gene expression, CTCF site disruption allowed for the formation of mCpG and H3mK9 methylation at the Qp initiation site (Fig. 7). These finding strongly suggest that CTCF contributes to the establishment and maintenance of an epigenetic pattern at Qp which is required for consistent expression of EBNA1 and episomal persistence in 293 cells pools. These findings also suggest that CTCF provides a barrier function that normally prevents Fp activation (upstream) and Qp silencing (downstream) during latent infection (Fig. 7D).

CTCF has been implicated in several gene regulatory and chromatin organizing activities [52], [53]. At the H19/Igf2 imprinted loci, CTCF functions as an enhancer blocker [62]. At the paternal allele, DNA methylation prevents CTCF binding, and allows enhancer activation of the Igf2 promoter. In contrast, at the unmethylated maternal allele, CTCF binds to a cluster of sites and prevents enhancer activation of the Igf2 promoter. Our findings here and previously [55] suggest that CTCF may have enhancer blocking activity at the sites surrounding OriP (e.g. the 5′ site at ∼6 kb and the 3′ site at 10 kb on the EBV genome). These sites are positioned to physically block OriP interactions with LMP1/2 and Cp, respectively. Interestingly, we found that CTCF binding at the 5′ site (∼6 kb on EBV) is reduced in type I latency where the sites have been partially subject to CpG DNA methylation. Thus, latency type-specific DNA methylation patterns may regulate CTCF binding at some regulatory regions. Conversely, CTCF binding may prevent DNA methylation at other regulatory elements. The region surrounding the Qp initiation site has been shown to lack CpG methylation in all latency types [43]. The CTCF site we identified may function to prevent the spread of CpG methylation which is normally elevated in the regions upstream of Qp (BFLF1 ORF) in both type I and type III latency. Disruption of the CTCF site caused a significant increase in CpG methylation immediately over the Qp initiation site (Fig. 7A). Thus, an essential function of CTCF may be to prevent CpG methylation at the Qp initiation site. This is consistent with the observation that CpG methylation is never detected at Qp in any EBV latency type [43].

Chromatin boundary factors, like CTCF, are thought to prevent the spread of processive histone modifications [48]. Our data suggests that CTCF has chromatin boundary activity at Qp that prevents inactivating heterochromatin, like H3mK9 from invading Qp. Elevation in H3mK9 at Qp was evident at 4 and 8 weeks post-transfection in ΔCTCF, suggesting that this mark is the first to cross into the Qp promoter region. At 16 weeks, mCpG is highly elevated at Qp, thus following H3mK9 and more completely silencing Qp transcription. Remarkably, the CTCF boundary functions in the reciprocal direction since the loss of CTCF leads to elevated transcription of Fp and BFLF1, along with a corresponding increase in histone H3mK4, and decrease in the normal H3mK9 and CpG methylation. It remains unclear what drives each modification at the specific sites. Our data strongly suggests that H3mK9 precedes the formation of mCpG, and that CTCF helps keep each modification in its proper place. Precisely what sets the pattern on each side of CTCF is not clear. CTCF may function in the recruitment of RNA polymerase II to Qp, and this may help to establish the correct orientation of the chromatin boundary at Qp. Loss of this boundary function leads to the loss of Qp transcription and the inappropriate regulation of other viral genes. CTCF also affected EBNA1 binding to Qp in vivo (Fig. 4D), raising the possibility that EBNA1 may also contribute to some of these changes in chromatin and transcription at Qp. Furthermore, it is not known if CTCF or EBNA1 provide additional structural features that directly affect these other promoters, or if these are indirect effects of altering EBNA1 levels in the 293 cell model of latency

DNA methylation, like histone modifications, can also spread across chromosomal regions to alter gene expression programs [63]. Epigenetic silencing due to promoter CpG methylation commonly arises at sights that have been actively repressed by histone deacetylation and K9 trimethylation. The mechanisms that restrict the drift of CpG methylation have not been completely elucidated. Our findings provide clear genetic evidence that CTCF can prevent the encroachment of CpG methylation at the Qp promoter of EBV. Our study shows that CpG methylation arises only after multiple generations at a region that is initially euchromatic and transcriptionally active. In the absence of CTCF, transcription initiation favors an alternative upstream promoter, Fp, which may prevent Qp utilization. Thus, CTCF may also facilitate promoter selection perhaps through its reported ability to interact with RNA polymerase II [64]. While the precise mechanism through which CTCF directs promoter selection and maintains chromatin boundaries remains to be discovered, our findings clearly indicate that CTCF provides an essential function in maintaining the epigenetic patterns at Qp. Our findings also indicate that protection of Qp by CTCF is essential for EBV genome stability during long-term latent infection. These findings also provide a framework for understanding the role of CTCF at other viral and cellular genes where protection from epigenetic drift and transcription silencing is critical for stable gene expression programs.

## Materials and Methods

### Cells

D98/HR1, HeLa and 293 cells were cultured in Dulbecco's modified Eagle's medium supplemented with 10% fetal bovine serum and antibiotics in a 5% CO_2_ incubator at 37°C. EBV positive Mutu I, Mutu-lymphoblastoid cell lines (Mutu-LCL), Sav I and Sav III cells were cultured in suspension in RPMI 1640 medium supplemented with 10% fetal bovine serum and antibiotics in a 5% CO_2_ incubator at 37°C. Mutu-LCL were established by primary infection of peripheral blood mononuclear cells (PBMCs) with EBV virions derived from stimulated Mutu I cells.

### Bacmids, plasmids and recombinant proteins

EBV bacmid was a generous gift of Dr. H. –J. Delecluse [65]. Mutations in EBV bacmid were generated by recombineering using the GalK marker gene insertion and negative selection method for its substitution as described previously (http://recombineering.ncifcrf.gov/) [56]. The GalK gene was recombined into the Qp region at EBV coordinates 49927–50185. The CTCF site at 50082 ggtcgctagatggcgcgggtgagg was mutated by single substitutions to ggtTgTtaTatTTTAcgggtgagg. The ΔEBNA1 binding site at 50142 gaaaag[gcgggatagcgtgcgctaccggatggcgggtaatacatgct]atccttaca was mutated by deletion and substitution with gaaag[tgcttgaaaaggcgcgg]atccttaca. Plasmid containing the Qp region (49712–50250) was subcloned by PCR into pBKSII using Asp718 and HinDIII sites. Recombinant human CTCF protein was expressed as an N-terninal hexa-histidine tagged fusion protein from a baculovirus expression virus in sf9 cells, as described previously[55].

### Chromatin Immunoprecipitation (ChIP) assay

ChIP assay followed the protocol provided by Upstate Biotechnology, Inc., with minor modifications as previously described [54]. Additional modifications are as follows. DNAs were sonicated to between 200- and 350-bp DNA fragments on a Diagenode Bioruptor according to manufacturer's protocol, and real-time PCR was performed with SYBER green probe in an ABI Prism 7900 using 1/100 to 1/2,500 of the ChIP DNA according to manufacturer's specified parameters. Primer sequences for the EBV genome array are available upon request. Antibodies for H3 me2 K4, H3 me3 K9, CTCF, were purchased from Upstate Biotechnology. Primary antibodies to EBNA1 (Advanced Biotechnologies, Inc.), CTCF (Millipore), GFP (Santa Cruz Biotecnology) and PCNA (Santa Cruz Biotecnology) were used according to manufacturer's specifications

### Electrophoretic mobility shift assay

EMSA assays with CTCF were described previously [55]. In a 20 µl reaction purified CTCF (∼100 ng) was added to a reaction mixture containing 0.5 µg poly(dI–dC), 5% glycerol, 0.1 mM ZnSO_4_, and 10,000 cpm of ^32^P-labeled DNA probe (∼0.1 ng). Reaction mixtures were incubated for 30 min at 25°C, electrophoresed in a 5% nondenaturing, polyacrylamide gel at 110 V, and visualized by PhosphorImager.

### DNase I footprinting analysis

DNase I footprinting was performed as described previously [66]. 5′-end labeled DS probe was generated using 30 µCi of [−^32^P]dATP (6,000 Ci/mmol; Perkin-Elmer) and 2 U of Klenow fragment (Roche) for 30 min at 25°C. Purified proteins were incubated in a reaction mixture containing 1XPBS, 5 mM MgCl_2_, 0.1 mM ZnSO_4_, 1 mM dithiothreitol, 0.1% NP-40, 10% glycerol, 1 µg bovine serum albumin, 0.4 µg poly(dI–dC), and 10,000 cpm of ^32^P-labeled probe. The protected probe was digested with different dilutions of DNase I (Sigma) and purified by phenol-chloroform extraction following proteinase K digestion. The DNA samples were then electrophoresed on a 7% denaturing, polyacrylamide sequencing gel at 33 mA and visualized by PhosphorImager.

### RNA extraction and RT-PCR

RNA was extract from 5×10^6^ cells using Qiagen RNA extraction Kit according to manufacturer's protocol (Qiagen). After the extraction the RNA was incubated with 2 U DNAse I at 37°C for 30 minutes, following by the inactivation of the enzyme at 65°C for 10 minutes. The RNA was quantified and 2 µg of RNA was reverse transcribed using Super Script II Reverse Transcriptase from Invitrogen. 50 ng of cDNA was then analyzed by real time or conventional PCR. Primer sequences used for real time and conventional PCR are listened in tables 2 and 3 (Tables S2 and S3).

### EBV genome copy number quantification

For EBV genome quantification Namalwa titration was used, assuming that Namalwa cell lines contain 2 copies of EBV genome and each cell contain 6.6×10^−12^ g of DNA. Namalwa cells and EBV positive cells were lysed in SDS lysis buffer (20 mM Tris pH 8, 4 mM EDTA, 20 mM NaCl, 1% SDS) following by incubation with Proteinase K for 2 h at 50°C. The DNA was extracted by phenol-chloroform extraction and precipitated by ethanol. Titration of 6.6×10^−7^–6.6×10^−12^ g of Namalwa DNA were used to obtain a calibration curve using primers for EBV genome (48779 – 48834) and β-Actin. 6.6×10^−9^ g of DNA from EBV positive cells were then analyzed by real time PCR for EBV genome copy quantification. Primer sequences used for real time PCR were listed in Tables S2 and S3.

### Methylated-DNA immunoprecipitation

10×10^6^ cells were resuspended in Lysis Buffer (20 mM Tris pH 8, 4 mM EDTA, 20 mM NaCl, 1% SDS) plus 0.7 µg/µl Proteinase K and incubated at 50°C overnight. The DNA was extracted by twice phenol-chloroform extraction followed by ethanol precipitation. The DNA was resuspended in 300 µl of TE buffer containing 20 µg/ml RNAse A and incubated at 37°C for 1 hr followed by DNA sonication to between 750 – 500 bp and DNA purification by phenol-chloroform extraction and ethanol precipitation. 8 µg of DNA were resuspended in Immunoprecipitation Buffer, IP buffer, [10 mM Na-Phosphate pH 7, 140 mM NaCl, 0.05% Triton X-100, and Proteinase inhibitor cocktail (Sigma)], denaturated at 95°C for 10 min and incubated with 5 µg of 5-methylCytodine antibody (Abcam) or 5 µg mouse IgG (Upstate), overnight at 4°C. The immunocomplexes were precipited by adding 50 µl of Protein G Dynabeads (Invitrogen) for 2 hr at 4°C. The beads were collected by a magnetic rack and washed twice for 10 min with 1 ml of IP buffer. The DNA was eluted by incubating the beads with 250 µl of Proteinase digestion buffer (50 mM Tris pH 8, 10 mM EDTA, 0.5% SDS, 0.3 µg/µl Proteinase K) at 50°C for 3 hr with shacking. The DNA was then purified and then analyzed by real time PCR. Primer sequences are listed in Table S4. 1 µg of genomic DNA was used as Input material.

### Quantitation of episomal DNA

Viral episomal DNA was extracted using the Hirt lysis method [67] Briefly, Wt rescue and ΔCTCF 293 cells were pelleted by centrifugation and then resuspended in 800 µl Hirt's lysis buffer (0.6% SDS, 10 mM EDTA, 10 mM Tris-HCl, pH 7.4) containing 500 µg Proteinase K. The samples were incubated at 37°C for 1 h, followed by addition of 200 µl of 5 M NaCl and incubated at 37°C overnight. The samples were centrifuged at 14000 g for 20 minute and the supernatant was transferred in a new tube. DNA was then purified by phenol/chloroform/isoamyl alcohol extraction and ethanol precipitation. The DNA was resuspended in 50 µl of TE buffer and analyzed by real Time PCR by ΔΔCt method, using mitochondrial DNA as endogenous control and viral DNA extracted from Raji cell as a standard.

## Supporting Information

Table S1 CTCF binding site identified by ChIP assay
(0.04 MB DOC)

Table S2 Primer sequences for Real Time PCR
(0.06 MB DOC)

Table S3 Primer sequences for RT-PCR
(0.03 MB DOC)

Table S4 Real time Primer sequences for Qp region
(0.04 MB DOC)

Figure S1 Histone modifications and copy number of type I and type III EBV latency. A) Histone modification analysis of Qp region in different latency types. ChIP assay was performed with Sav I (type I) or Sav III (type III) using antibodies for H3me2K4, H3me3K9, CTCF and EBNA1 ChIP DNA was assayed by real time PCR using primer sets for a region from −5.8 kb to +2.7 kb of Qp. B) ChIP assay as in A but using IgG as control for non-specific binding. C) EBV genome copy number in EBV positive cells (Mut I, Mutu-LCL, Sav I, and Sav III) was quantified by real time PCR.
(5.07 MB TIF)

Figure S2 EBNA2 RNA expression and promoter utilization in different EBV cell lines. Real time PCR was used to validate the pattern of gene expression program I or III in EBV positive cells (Mutu I, Mutu-LCL, Sav I, and Sav III), by measuring Cp and Qp promoter utilization and EBNA2 expression assays, as indicated.
(0.70 MB TIF)

Figure S3 Lytic replication was assayed in Rescue or ΔCTCF 293 cells pool. Virus reactivation was evaluated by real time PCR measuring Zta mRNA expression in Rescue or ΔCTCF 293 cells pool. Sodium butyrate (NaB) and phorbol ester (TPA) was used to activate lytic replication from Wt rescue or ΔCTCF 293 cells pools as a positive control.
(2.85 MB TIF)

Figure S4 RNA expression and promoter utilization in GalK-Qp mutated bacmids. A) Quantitative RT-PCR was used to measure the abundance of EBNA2, EBNA3A and EBNA3C mRNA relative to bacmid GFP for Wt rescue or GalK-Qp bacmids in 293 cell pools. B) Same as in A, except mRNA for EBNA1-transcripts were measured relative to GFP. C) RT-PCR was measured for Wt rescue or GalK-Qp bacmids in 293 cell pools, as well as for type I (Mutu I) or type III (Mutu-LCL) controls. RNA was analyzed for initiation the junction specific transcripts QUK (Qp initiation), C_1_C_2_W_1_W_2_ (Cp initiation), W_0_W_1_W_2_ (Wp initiation), BFLF1 (lytic gene adjacent to Qp), UK (EBNA1 mRNA in both type I and type III), and control cellular GAPDH.
(4.37 MB TIF)

Figure S5 Change in Epigenetic patterns in Cp and Wp in mutated bacmids. A) The epigenetic pattern of Cp and Wp in Wt rescue and ΔCTCF bacmids in 293 cell pools was analyzed by MeDIp assay (A), H3me2K4 (B) and H3me3K9 (C) ChIp assay at 8 weeks (top panel) or 16 weeks (lower panel) after transfection.
(4.54 MB TIF)

Figure S6 Control ChIP for Figure 7 using non-specifc IgG is shown for MeDIp (A) or ChIP (B) assays at the Qp, the Cp and the Wp loci.
(3.04 MB TIF)

Figure S7 Normalization of RNA for RT-PCR. A) GFP mRNA was normalized relative to cellular Actin at 8 and 16 weeks after transfection into 293 cells for Wt rescue (black) and ΔCTCF (red) bacmids. B) GFP mRNA was normalized relative to the DNA copy number for each bacmid with same samples as shown in panel A.
(0.14 MB TIF)
