# Supplementary material for: CTCF Prevents the Epigenetic Drift of EBV Latency Promoter Qp
Source: PLoS Pathog. 2010 Aug 12;6(8):e1001048. doi: 10.1371/journal.ppat.1001048 (PMC2921154; doi:10.1371/journal.ppat.1001048)
Supplement: Table S1 — CTCF binding site identified by ChIP assay (0.04 MB DOC) [file ppat.1001048.s001.doc]

| **EBV region** | **Mutu occupancy (%)** | **LCL occupancy (%)** | **Hypotetical CTCF binding site** |
| --- | --- | --- | --- |
| 6301-6800 | 0.05 | 0.66 | **6485-6504** |
| 10351-10800 | 0.8 | 0.54 | **10515-10560*** |
| 10801-11050 | 0.19 | 0.03 |
| 11051-11250 | 0.22 | 0.05 | **11159-11178** |
| 35551-36000 | 1.3 | 1.05 | **35974-35993** |
| 36001-36450 | 0.8 | 0.68 |
| 40001-40500 | 0.44 | 0.43 | **40634-40652**  **40754-40773** |
| 40501-40950 | 0.58 | 0.30 |
| 49050-49500 | 0.31 | 0.24 | **49739-49758** |
| 49501-49950 | 0.50 | 0.32 |
| 49951-50400 | 0.85 | 0.40 | **50082-50102 **** |
| 64801-65250 | 1 | 0.06 | **63510-63529** |
| 67951-68400 | 0.63 | 0.20 | **67955-67974** |
| 143100-143550 | 0.40 | 0.43 | **143673-143692** |
| 143551-144000 | 1.2 | 1.3 |
| 144001-144451 | 0.45 | 0.3 |

**Table 1. CTCF binding site identified by ChIP assay**

* Confirmed in Chau CM, Zhang XY, McMahon SB, Lieberman PM (2006) Regulation of Epstein-Barr virus latency type by the chromatin boundary factor CTCF. J Virol 80: 5723-5732.

.

** Confirmed in this paper
