# Supplementary material for: CTCF Prevents the Epigenetic Drift of EBV Latency Promoter Qp
Source: PLoS Pathog. 2010 Aug 12;6(8):e1001048. doi: 10.1371/journal.ppat.1001048 (PMC2921154; doi:10.1371/journal.ppat.1001048)
Supplement: Table S2 — Primer sequences for Real Time PCR (0.06 MB DOC) [file ppat.1001048.s002.doc]

| **Transcript** | **Primer** | **Sequence (5'-3')** | **EBV coordinates** |
| --- | --- | --- | --- |
| EBNA1 WP/Cp | 5' primer | TGCCTGAACCTGTGGTTGG | **36134-36152** |
| EBNA1 Qp | 5' primer | GTGCGCTACCGGATGGC | **50152-50168** |
| EBNA1 WP/Cp/Qp | 3' primer | CATGATTCACACTTAAAGGAGACGG | **95664-95654/55361-55348** |
| FQUK | 5' primer | GGGTGAGGCCACGCTTT | **50099-50115** |
| FQUK | 3' primer | CAGGTCTACTGGCGGTCTATGAT | **55326-55304** |
| EBNA1 | 5' primer | GGTCGTGGACGTGGAGAAAA | **96778-96797** |
| EBNA1 | 3' primer | GGTGGAGACCCGGATGATG | **96845-96827** |
| EBNA 2 | 5' primer | GCTTAGCCAGTAACCCAGCACT | **35702-35709/36095-36109** |
| EBNA 2 | 3' primer | TGCTTAGAAGGTTGTTGGCATG | **36181-36160** |
| EBNA3A | 5' primer | GATGAGGCCTAAGCAAAGGTGTA | **82866-82888** |
| EBNA3A | 3' primer | GGCGTATTATCAGTGGGTGGAA | **82960-82923** |
| EBNA3C | 5' primer | TAATGCCACCACGCCAAA | **88958-88975** |
| EBNA3C | 3' primer | GGGCAGGTCCGTGAGAACT | **89013-88995** |
| BZLF1 | 5' primer | CTTGGCCCGGCATTTTCT | **90168-90165** |
| BZLF1 | 3' primer | ACGACGCACACGGAAACC | **90400-90383** |
| β-Actin | 5' primer | GGCTCACCACTGCAGAAATCA |  |
| β-Actin | 3' primer | TTATCTTGGAGGTCCCCT |  |
| GAPDH | 5' primer | TGGGCTACACTGAGCACCAG |  |
| GAPDH | 3' primer | GGGTGTCGCTGTTGAAGTCA |  |
| GFP | 5' primer | AGCAAAGACCCCAACGAGAA |  |
| GFP | 3' primer | GGCGGCGGTCACGAA |  |

**Table 2. Primer sequences for Real Time PCR**
