# Supplementary material for: CTCF Prevents the Epigenetic Drift of EBV Latency Promoter Qp
Source: PLoS Pathog. 2010 Aug 12;6(8):e1001048. doi: 10.1371/journal.ppat.1001048 (PMC2921154; doi:10.1371/journal.ppat.1001048)
Supplement: Table S3 — Primer sequences for RT-PCR (0.03 MB DOC) [file ppat.1001048.s003.doc]

| **Transcript** | **Primer** | **Sequence (5’ -3’)** | **EBV coordinates** |
| --- | --- | --- | --- |
| C1/C2 | 5’ primer | ATCTAAACCGACTGAAGAA | **11470-11479/11626-11635** |
| W0/W1 | 5’primer | GTCCACACAAATCCTAG | **14399-14410/14554-14558** |
| C/W | 3’primer | CCCTGAAGGTGAACCGCTTA | **14832-14813** |
| U | 5’primer | GATGAGCGTTTGGGAGAGCTGATTCTGCA | **55223-55251** |
| F | 5’primer | GGATCCGGAGGGGACCACTA | **49961-49980** |
| K | 3’primer | CATTTCCAGGTCCTGTACCT | **95698-95679** |
| BFLF1 | 5’primer | AAAGTCACGAGCGCAAAC | **46228-46211** |
| BFLF1 | 3’primer | TGAGGCTAGAGATCCAGG | **45829-45846** |
| GAPDH | 5’primer | TCACCACCATGGAGAAGGCT |  |
| GAPDH | 3’primer | GCCATCCACAGTCTTCTGGG |  |

**Table 3. Primer sequences for RT-PCR**
