# Supplementary material for: CTCF Prevents the Epigenetic Drift of EBV Latency Promoter Qp
Source: PLoS Pathog. 2010 Aug 12;6(8):e1001048. doi: 10.1371/journal.ppat.1001048 (PMC2921154; doi:10.1371/journal.ppat.1001048)
Supplement: Table S4 — Real time Primer sequences for Qp region (0.04 MB DOC) [file ppat.1001048.s004.doc]

| **Qp region** | **Primer** | **Sequence (5'- 3')** | **EBV coordinates** |
| --- | --- | --- | --- |
| - 1000 bp | 5' primer | TGACCAGAAGGAGAAGCATGTG | **48779 - 48800** |
| - 1000 bp | 3' primer | GCAGAGCCGCAGTTGGA | **48850 - 48834** |
| Fp | 5' primer | TCCCCAATGATGTTTTTCG | **49322 - 49340** |
| Fp | 3' primer | GCAGAACTGGGATGTCAGAA | **49386 - 49367** |
| Qp | 5' primer | GGCTCACGAAGCGAGAC | **49867-49883** |
| Qp | 3' primer | ACAGGACCTGCGTTATAGCC | **49931 - 49912** |
| + 800 | 5' primer | GAATCTCCGCGGCTTCTTC | **50755 - 50773** |
| + 800 | 3' primer | CCTATGGCCATTCTATCATGCA | **50823 - 50802** |

**Table 4. Real time Primer sequences for Qp region**
