# Supplementary figures and images for: CTCF Prevents the Epigenetic Drift of EBV Latency Promoter Qp
Source: PLoS Pathog. 2010 Aug 12;6(8):e1001048. doi: 10.1371/journal.ppat.1001048 (PMC2921154; doi:10.1371/journal.ppat.1001048)

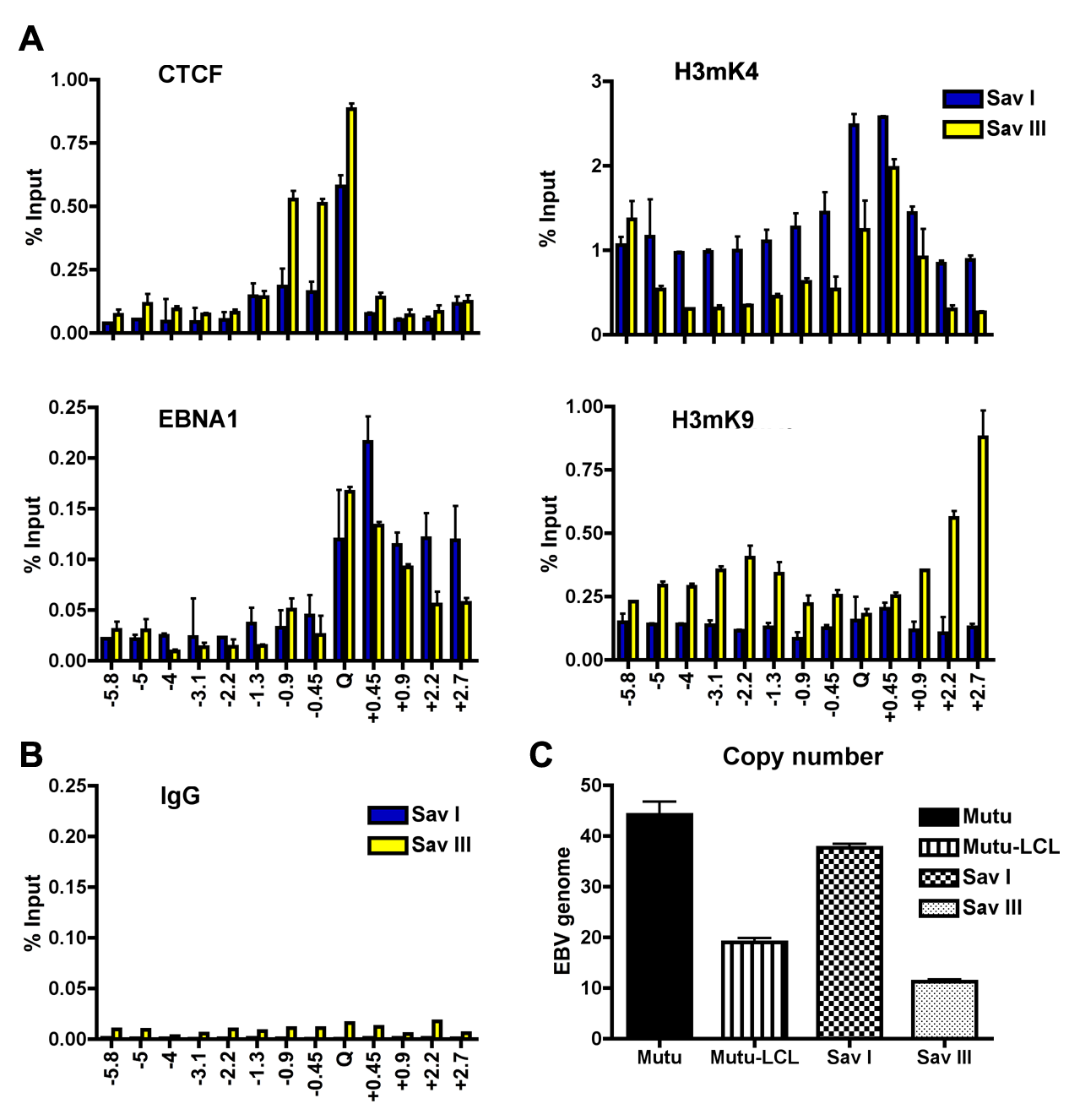

Supplement: Figure S1 — Histone modifications and copy number of type I and type III EBV latency. A) Histone modification analysis of Qp region in different latency types. ChIP assay was performed with Sav I (type I) or Sav III (type III) using antibodies for H3me2K4, H3me3K9, CTCF and EBNA1 ChIP DNA was assayed by real time PCR using primer sets for a region from −5.8 kb to +2.7 kb of Qp. B) ChIP assay as in A but using IgG as control for non-specific binding. C) EBV genome copy number in EBV positive cells (Mut I, Mutu-LCL, Sav I, and Sav III) was quantified by real time PCR. (5.07 MB TIF) [file ppat.1001048.s005.tif]

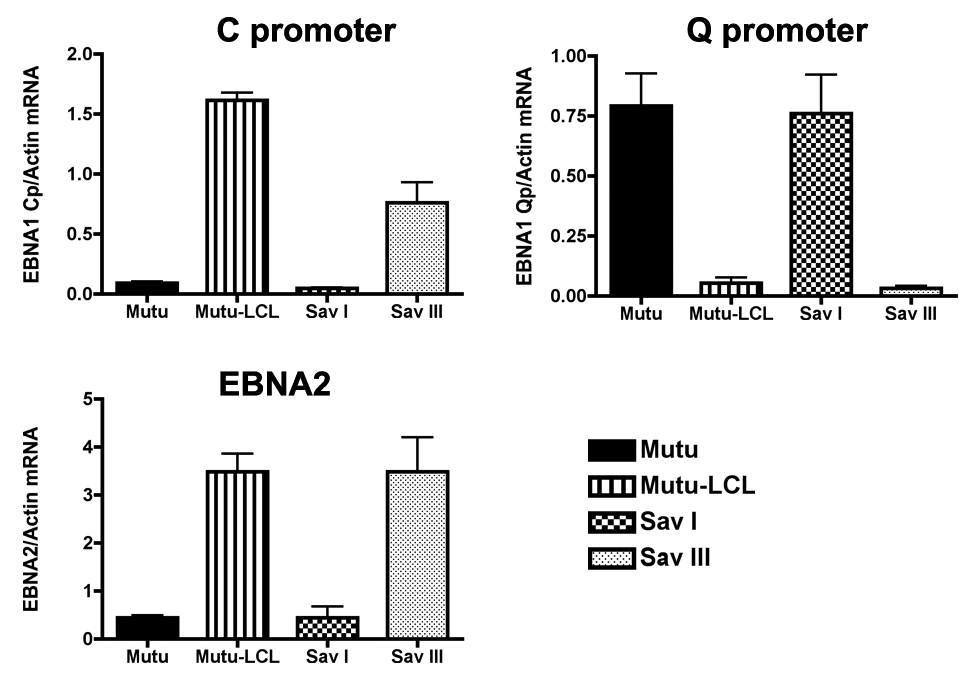

Supplement: Figure S2 — EBNA2 RNA expression and promoter utilization in different EBV cell lines. Real time PCR was used to validate the pattern of gene expression program I or III in EBV positive cells (Mutu I, Mutu-LCL, Sav I, and Sav III), by measuring Cp and Qp promoter utilization and EBNA2 expression assays, as indicated. (0.70 MB TIF) [file ppat.1001048.s006.tif]

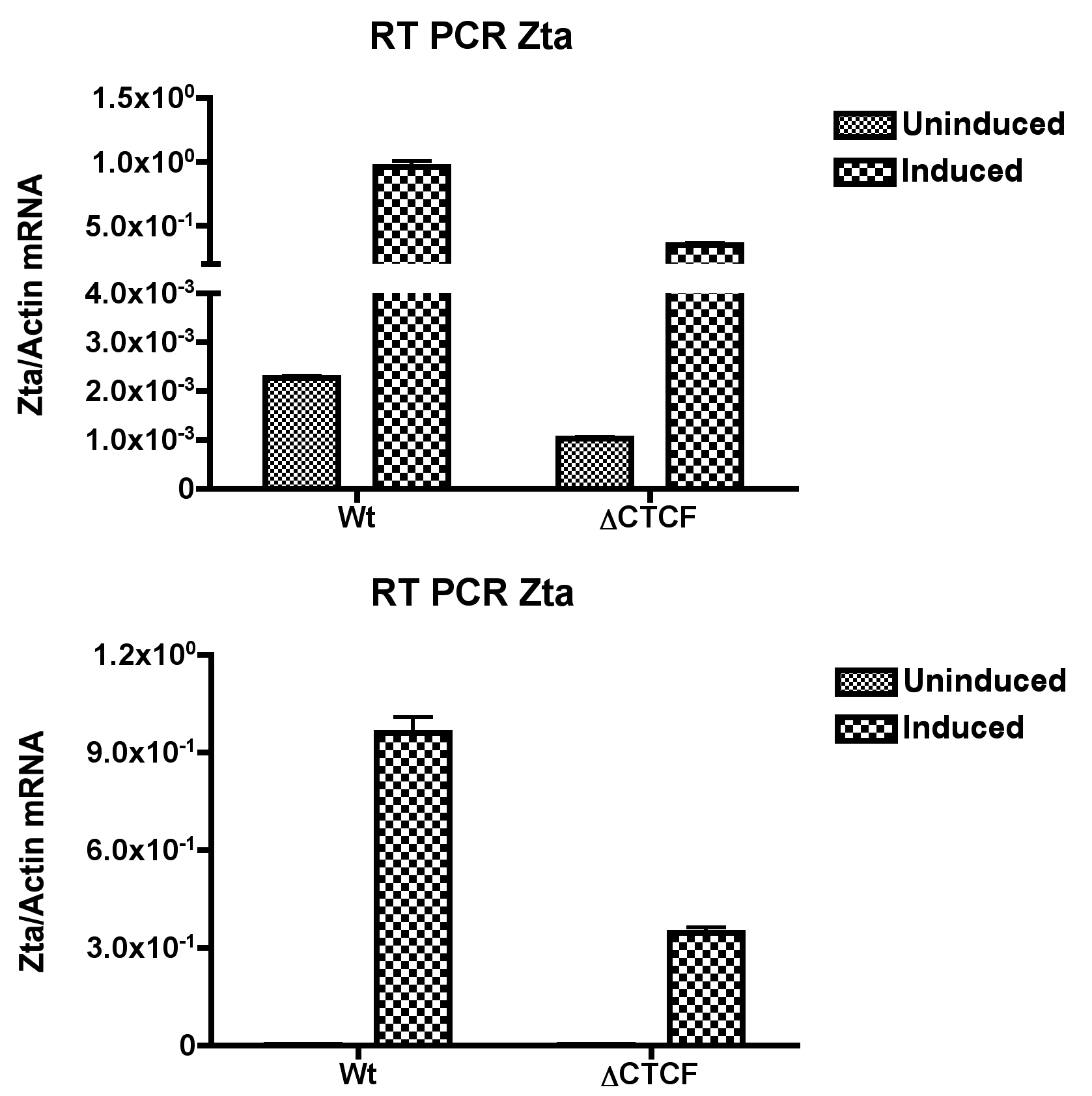

Supplement: Figure S3 — Lytic replication was assayed in Rescue or ΔCTCF 293 cells pool. Virus reactivation was evaluated by real time PCR measuring Zta mRNA expression in Rescue or ΔCTCF 293 cells pool. Sodium butyrate (NaB) and phorbol ester (TPA) was used to activate lytic replication from Wt rescue or ΔCTCF 293 cells pools as a positive control. (2.85 MB TIF) [file ppat.1001048.s007.tif]

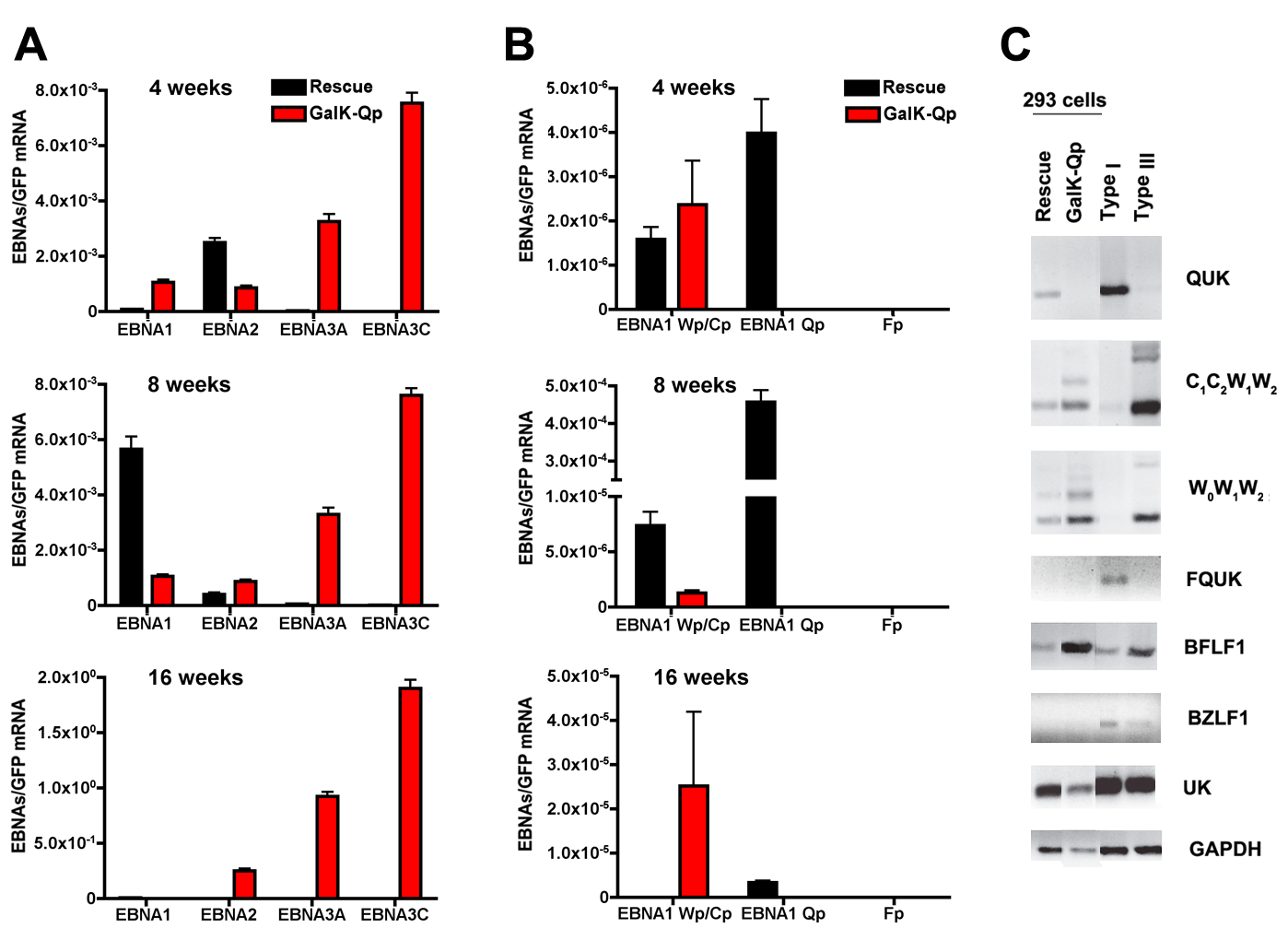

Supplement: Figure S4 — RNA expression and promoter utilization in GalK-Qp mutated bacmids. A) Quantitative RT-PCR was used to measure the abundance of EBNA2, EBNA3A and EBNA3C mRNA relative to bacmid GFP for Wt rescue or GalK-Qp bacmids in 293 cell pools. B) Same as in A, except mRNA for EBNA1-transcripts were measured relative to GFP. C) RT-PCR was measured for Wt rescue or GalK-Qp bacmids in 293 cell pools, as well as for type I (Mutu I) or type III (Mutu-LCL) controls. RNA was analyzed for initiation the junction specific transcripts QUK (Qp initiation), C1C2W1W2 (Cp initiation), W0W1W2 (Wp initiation), BFLF1 (lytic gene adjacent to Qp), UK (EBNA1 mRNA in both type I and type III), and control cellular GAPDH. (4.37 MB TIF) [file ppat.1001048.s008.tif]

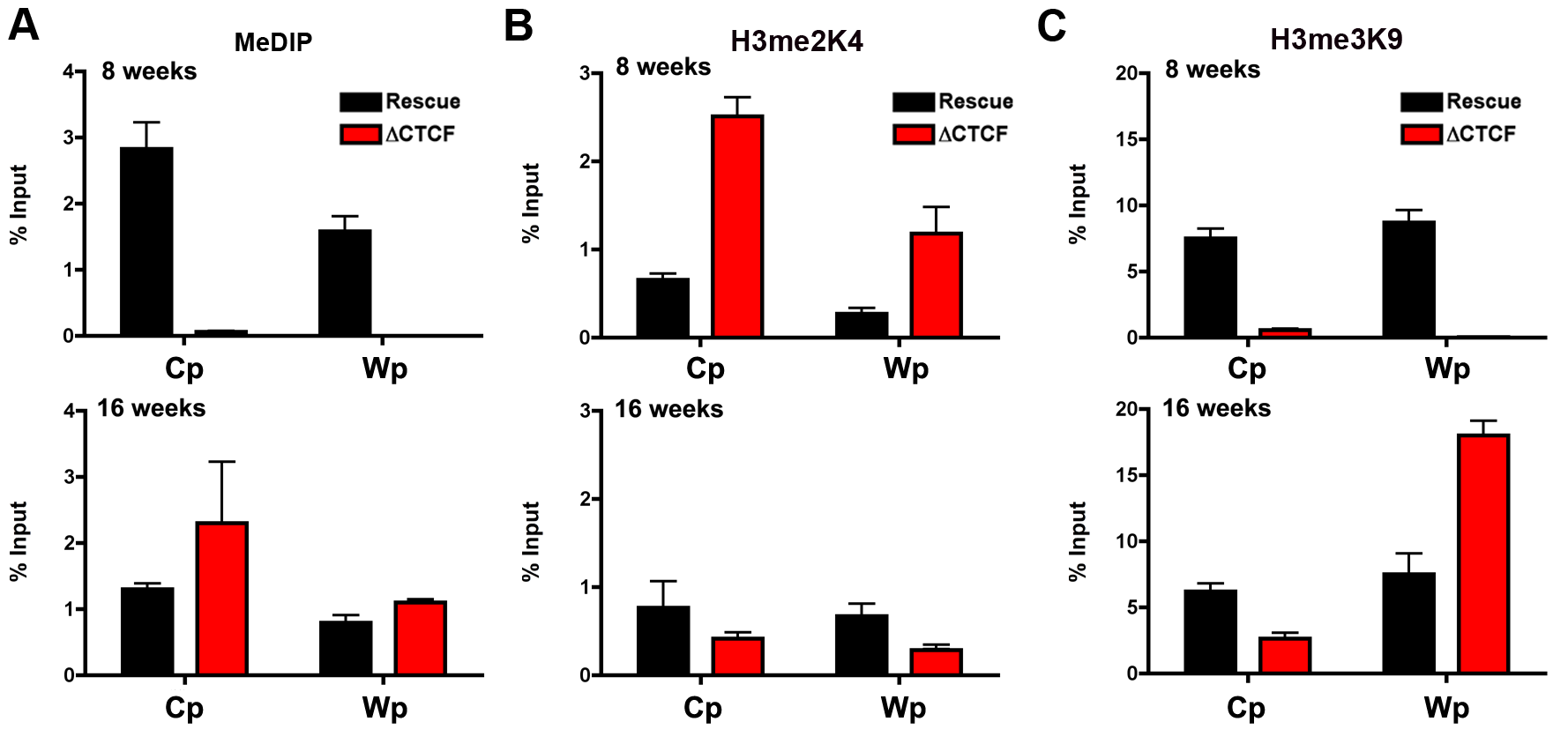

Supplement: Figure S5 — Change in Epigenetic patterns in Cp and Wp in mutated bacmids. A) The epigenetic pattern of Cp and Wp in Wt rescue and ΔCTCF bacmids in 293 cell pools was analyzed by MeDIp assay (A), H3me2K4 (B) and H3me3K9 (C) ChIp assay at 8 weeks (top panel) or 16 weeks (lower panel) after transfection. (4.54 MB TIF) [file ppat.1001048.s009.tif]

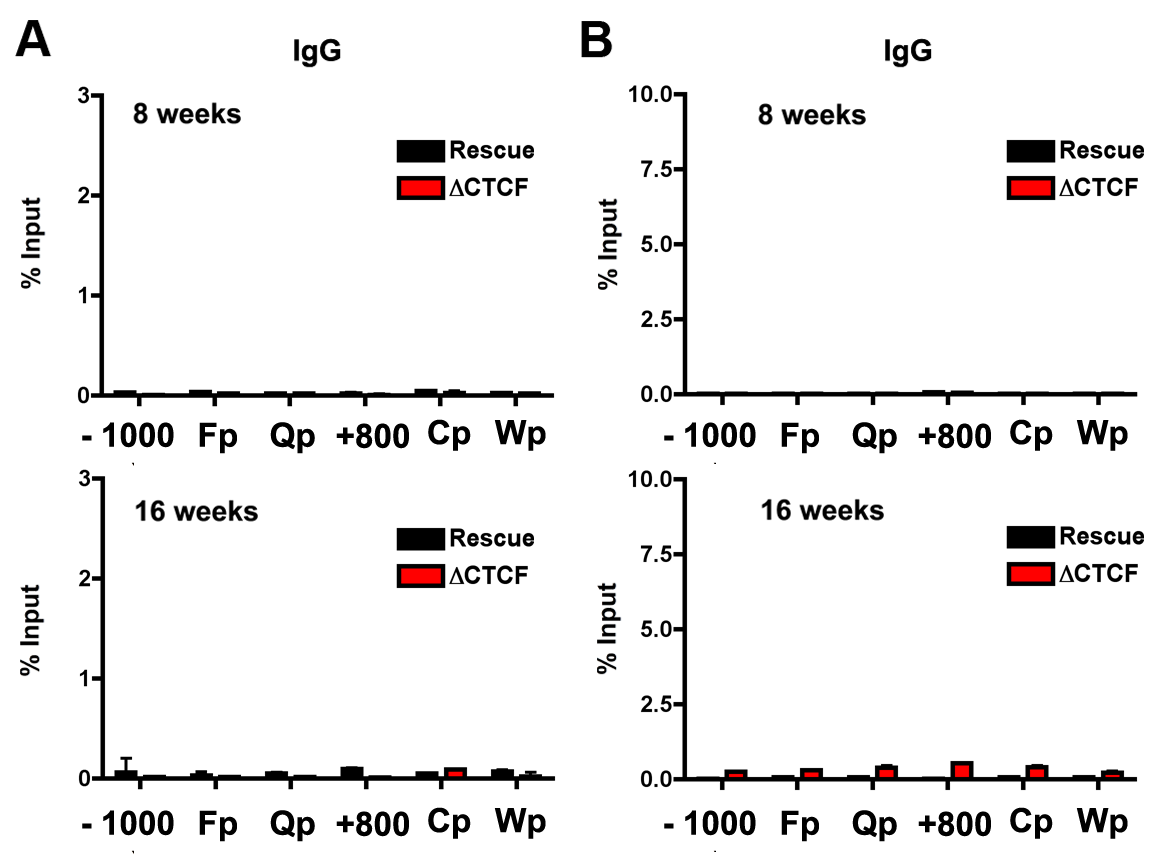

Supplement: Figure S6 — Control ChIP for Figure 7 using non-specifc IgG is shown for MeDIp (A) or ChIP (B) assays at the Qp, the Cp and the Wp loci. (3.04 MB TIF) [file ppat.1001048.s010.tif]

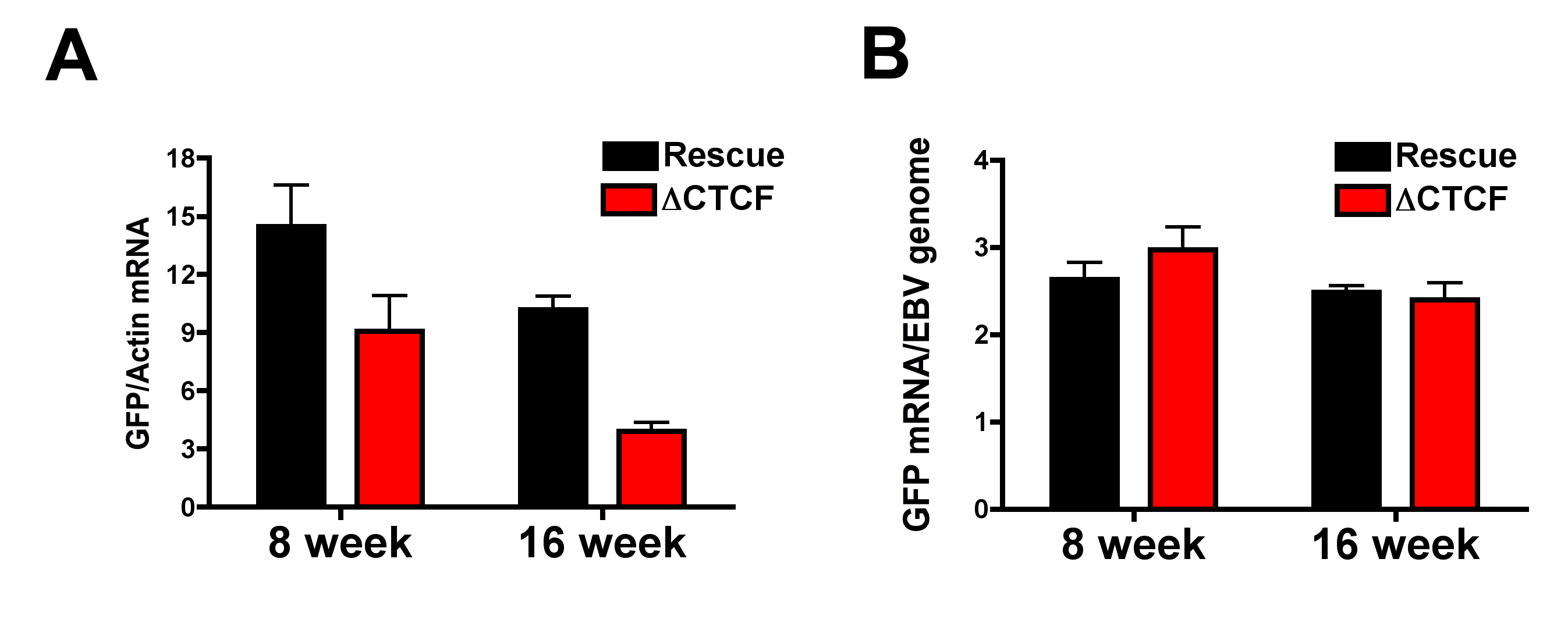

Supplement: Figure S7 — Normalization of RNA for RT-PCR. A) GFP mRNA was normalized relative to cellular Actin at 8 and 16 weeks after transfection into 293 cells for Wt rescue (black) and ΔCTCF (red) bacmids. B) GFP mRNA was normalized relative to the DNA copy number for each bacmid with same samples as shown in panel A. (0.14 MB TIF) [file ppat.1001048.s011.tif]
